# Global research priorities on COVID-19 for maternal, newborn, child and adolescent health

**DOI:** 10.7189/jogh.11.04071

**Published:** 2021-11-20

**Authors:** COVID-19 Research Prioritization Group on MNCAH, Nawshad Uddin Ahmed ASM, Abdellatif Maamri, Adegoke G Falade, Adejumoke Idowu Ayede, Adnan Bhutta, Ajay Gambhir, Alfredo Tagarro, Ali Abdelmegeid, Ali Reza Ahmadi, Aluísio J D Barros, Amha Mekasha, Anantha Kumar Srinivasaiyer, André Ricardo Araujo da Silva, Andreas Schultz, Batool Fatima, Bishnupada Dhar, Brian Magowan, Bridget Wills, Camille Raynes-Greenow, Caroline Homer, Carolyn Maclennan, Catherine Ward, Daniel Martinez Garcia, David Ross, David Murdoch, Deborah Joy Wilson, Ebun Adejuyigbe, Ecaterina Stasii, Elaine Scudder, Emma Sacks, Eric D McCollum, Fernando Althabe, Fiona Russell, GS Kumar, Halvor Sommerfelt, Hamish Graham, Hannah Blencowe, Hannah Tappis, Haroon Saloojee, Hesham Abdussalam Ben Masaud, Hiresh Tiwary, Ifeyinwa Asiodu, James B Newton, Jessica Bourdaire, Joel Amwe Adze, Jose Martines, Juan M Lozano, Judd Walson, Judith Rankin, Karel Allegaert, Karell G Pellé, Karen Edmond, Katayoun Rabiei, Kathleen M Rasmussen, Khalid Yunis, Laura Ferguson, Leith Greenslade, Lilian Kiapi, Lisa Noguchi, Louis Bont, Louise T Day, Lynne Mofenson, Maher Aboumayaleh, Majda Aquzouz, Mamdouh Wahba, Mari Nagai, Marian Knight, Marina Melkumova, Mariyam Jenyfa, Mark I Neuman, Martin Meremikwu, Mary Kinney, Michael Gravett, Michael T Hawkes, Michel Pacqué, Michele Walsh, Michelle K McGuire, Nagiba AAbdulghani AlShawafi, Najwa Khuri-Bulos, Naveen Thacker, Nigel Rollins, Niranjan Kissoon, Olena Starets, Olivier Picone, Olufemi T Oladapo, Omer Erdeve, P Brian Smith, Philippe Van de Perre, Praveen Kumar, Punam Mangtani, Qalab Abbas, Rabeya Khatoon, Rajiv Bahl, Rakesh Lodha, Rebecca Grais, Rebecca Richards-Kortum, Reeta Rasaily, Richmond Aryeetey, Robert Pattinson, Roberta Petrucci, Rodolfo Rossi, Ryan M Pace, Sachiyo Yoshida, Salimah R Walani, Sanjeeva SP Godakandage, Sarah Bauler, Sarah S Comstock, Saurav Basu, Senait Kebede, Senjuti Saha, Shinjini Bhatnagar, Shoo Kim Lee, Shuchita Gupta, Simon Nyovuura Antara, Soo Downe, Stephen Freedman, Stephen J Lye, Suellen Miller, Suha Sulimani, Sylvia H Ley, Tara D Mangal, Tina Lavin, Ting Shi, Todd A Florin, Ulrik Kræmer Sundekilde, Valentina Baltag, Veronica Valdes, William Cherniak, Yasir Bin Nisar, Zainularab Zohra Shamszai, Zohreh Sadat Navabi

**Affiliations:** 1 Dhaka Shishu (Children) Hospital & Child Health Research Foundation (CHRF), Dhaka, Bangladesh; 2 University Mohammed first Oujda, Morocco; 3 College of Medicine, University of Ibadan, Ibadan, Nigeria; 4 University of Ibadan & University College Hospital Ibadan, Nigeria; 5 Department of Pediatrics, University of Maryland School of Medicine, US; 6 Vaccine India Trust, National Newborn Foundation, Delhi Medical Association; 7 Pediatrics Department Hospital Universitario Infanta Sofía, Unidad Pediátrica de Investigación y Ensayos Clínicos (UPIC) Instituto de Investigación 12 de Octubre, Universidad Europea de Madrid; 8 Allied Experts for Health Systems; Egypt; 9 Pediatric Cardiovascular Research Center, Cardiovascular Research Institute, Isfahan University of Medical Sciences, Isfahan, Iran; 10 Universidade Federal de Pelotas, Pelotas, Brasil; 11 Department of Pediatrics and Child health, Addis Ababa University, Ethiopia; 12 Swami Vivekananda Youth Movement, Karnataka, India; 13 Federal Fluminense University, Materno Infantil Department, Niterói, Brazil; 14 Institute for Hygiene and Public Health Section Global Health, University of Bonn, Germany; 15 Department of Mental Health and Substance Use, World Health Organization; 16 Independent Consultant & Researcher; 17 NHS Borders, Scotland; 18 Oxford Centre for Global Health Research, University of Oxford, UK and Oxford University Clinical Research Unit, Ho Chi Minh City, Vietnam; 19 Associate Prof, The University of Sydney, Sydney School of Public Health, Australia; 20 Burnet Institute, Australia; 21 Northern Territory Australia; 22 University of Cape Town, South Africa; 23 Médecins Sans Frontières (MSF) – Women and Children’s Health Unit, Medical Department, Operational Centre Geneva, SWITZERLAND; 24 Department of Maternal, Child and Adolescent Health and Ageing, World Health Organization; 25 University of Otago, Christchurch, New Zealand; 26 United Nations World Food Programme; 27 Obafemi Awolowo University, Ile-Ife, Nigeria; 28 State Medical and Pharmaceutical University after Nicolae Testemitanu, Moldova; 29 Inter-Agency Working Group on Reproductive Health in Crises (IAWG) / International Rescue Committee; 30 Department of International Health, Johns Hopkins School of Public Health, USA; 31 Global Program in Respiratory Sciences, Eudowood Division of Pediatric Respiratory Sciences, Department of Pediatrics, Johns Hopkins School of Medicine, USA; 32 Department of Sexual and Reproductive Health, World Health Organization; 33 The University of Melbourne; and Murdoch Children's Research Institute, Australia; 34 Swami Vivekananda Youth Movement, Saragur, Mysuru District, Karnataka, India; 35 Centre for International Health, University of Bergen and Cluster for Global Health, Division for Health Services, Norwegian Institute of Public Health, Norway; 36 University of Melbourne, Murdoch Children's Research Institute, Royal Children's Hospital Melbourne, Australia; 37 London School of Hygiene & Tropical Medicine, UK; 38 Jhpiego – an affiliate of Johns Hopkins University Baltimore, Maryland, USA and Department of International Health, Johns Hopkins Bloomberg School of Public Health, Baltimore, Maryland, USA; 39 University of the Witwatersrand, Johannesburg, South Africa; 40 Libyan medical board, Libyan general practice society, Libyan emergency medicine association, Primary health care institute, Libya; 41 Society for Applied Studies, India; 42 University of California, San Francisco (UCSF) School of Nursing, San Francisco, US; 43 University of Pittsburgh; Lean Med, LLC, USA; 44 United Nations World Food Programme, Nutrition Research Officer; 45 Kaduna State University, Kaduna, Nigeria; 46 CISMAC, University of Bergen, Norway; 47 Division of Medical and Population Health Sciences Education and Research Department of Translational Medicine Herbert Wertheim College of Medicine Florida International University, USA; 48 University of Washington, USA; 49 Population Health Sciences Institute, Newcastle University, UK; 50 Erasmus MC Rotterdam, Netherlands; 51 Foundation for Innovative New Diagnostics, Switzerland; 52 Cornell University, Ithaca, New York, USA; 53 American University of Beirut, Lebanon; 54 Institute on Inequalities in Global Health, University of Southern California, USA; 55 Every Breath Counts Coalition; 56 International Rescue Committee; 57 Johns Hopkins Bloomberg School of Public Health, USA; 58 UMC Utrecht, Netherland; 59 Maternal & Newborn Health Group, Centre for Maternal Adolescent Reproductive & Child Health (MARCH), London School of Hygiene & Tropical Medicine (LSHTM), UK; 60 Elizabeth Glaser Pediatric AIDS Foundation; 61 Aga Khan Health Services, Syria; 62 Sexual and Reproductive Health Advisor, International Committee of the Red Cross; 63 Egyptian Society for Adolescent Medicine, Egypt; 64 Bureau of International Health Co-operation National Center for Global Health & Medicine (NCGM), Japan; 65 Professor of Maternal and Child Population Health, National Perinatal Epidemiology Unit, Nuffield Department of Population Health, University of Oxford, UK; 66 Arabkir Medical Centre-Institute of Child and Adolescent Health, National Institute of Health, Armenia; 67 Health Protection Agency, Ministry of Health, Maldives; 68 Division of Emergency Medicine, Boston Children's Hospital, Department of Pediatrics, Harvard Medical School, USA; 69 University of Calabar, Nigeria; 70 School of Public Health, University of the Western Cape, South Africa; 71 Global Health University of Washington Seattle, WA (USA); 72 Department of Pediatrics, University of Alberta, Canada; 73 Child Health Team Lead, JSI Research & Training Institute, Inc, USA; 74 Case Western Reserve University, Cleveland OH USA; 75 Margaret Ritchie School of Family and Consumer Sciences, University of Idaho, USA; 76 Medical & Health Science Faculty, Sanaa University, Yemen; 77 University of Jordan, Amman, Jordan; 78 Deep Children Hospital & Research Center, India and International Pediatrics Association; 79 Children’s and Women’s Global Health, UBC and BCCH Professor in Acute and Critical Care, Global Child Health, BCCH Research Institute, Global Sepsis Alliance, Vancouver; 80 National Medical University, Department of Pediatrics, Odessa, Ukraine; 81 Hôpital L Mourier, Colombes, France, University of Paris; 82 Ankara University School of Medicine, Department of Pediatrics, Division of Neonatology, Ankara, Turkey; 83 Duke University Medical Center, USA; 84 University Montpellier, INSERM, Establishment Français du Sang, Antilles University; CHU Montpellier, France; 85 Post Graduate Institute of Medical Education and Research, Chandigarh; 86 Department of Infectious Disease Epidemiology London School of Hygiene and Tropical Medicine, UK; 87 Department of Pediatrics and Child Health, Aga Khan University, Karachi, Pakistan; 88 Free-lance consultant RMNCAH; 89 Department of Pediatrics, All India Institute of Medical Sciences, New Delhi, India; 90 Epicentre; 91 Malcolm Gillis University Professor, Rice 360 Institute for Global Health, Rice University, Houston, TX (USA); 92 Indian Council of Medical Research, New Delhi, India; 93 University of Ghana School of Public Health Legon, Accra, Ghana; 94 SAMRC/UP Maternal and Infant Health Care Strategies; 95 Medecins Sans Frontieres – International Pediatric Working group; 96 International Committee of the Red Cross (ICRC); 97 Global Programs, March of Dimes, USA; 98 Consultant Community Physician Family Health Bureau/ Ministry of Health, Sri Lanka; 99 Senior Advisor, DME & Research, World Vision International; Global Health Instructor, Appalachian State University, USA; 100 Michigan State University, USA; 101 Maulana Azad Medical College, New Delhi, India; 102 Adjunct Associate Professor of Global Health, Emory University, USA; 103 Child Health Research Foundation, Bangladesh; 104 Translational Health Science and Technology Institute (DBT, Govt of India), Delhi, India; 105 University of Toronto, Canada; 106 African Field Epidemiology Network; 107 University of Central Lancashire, UK; 108 University of Calgary, Canada; 109 University of Toronto, Alliance for Human Development, Lunenfeld-Tanenbaum Research Institute, Sinai Health and University of Toronto, Canada; 110 University of California, San Francisco, USA; 111 Ministry of Health, Saudi Arabia; 112 Tulane University School of Public Health & Tropical Medicine, USA; 113 Imperial college, London; 114 Usher Institute, University of Edinburgh; 115 Ann and Robert H Lurie Children's Hospital of Chicago & Northwestern University Feinberg School of Medicine, UK; 116 Deptartment of Food Science, Faculty of Technical Sciences, Aarhus University Denmark, Denmark; 117 Universidad Católica de Chile, Chile; 118 Bridge to Health Medical and Dental Canada & USA and University of Toronto, Department of Family and Community Medicine, Division of Emergency Medicine, USA; 119 Obstetrician, Gynecologist, RMNCAH consultant, The Netherlands

## Abstract

**Background:**

This research prioritization aimed to identify major research gaps in maternal, newborn, child and adolescent health (MNCAH) to help mitigate the direct and indirect effects of the COVID-19 pandemic.

**Methods:**

We adapted the Child Health and Nutrition Research Initiative methodology. We defined scope, domains, themes and scoring criteria. We approached diverse global experts via email to submit their research ideas in MNCAH and MNCAH-related cross-cutting/health systems area. We curated the research ideas as research questions (RQs) and sent them to the consenting experts for scoring via the online link. For each RQ, the research priority score (RPS) was calculated as an average of individual criterion scores and ranked based on RPS in each area.

**Results:**

We identified top-ranked 10 RQs in each maternal, newborn, and child and adolescent health and 5 in the cross-cutting/health systems area. In maternal health, indirect effects on care, measures to improve care, health risks and outcomes, and preventing and managing SARS-CoV-2 infection/COVID-19 disease were priority RQs. In newborn health, clinical characterization and managing SARS-CoV-2 infection/COVID-19 disease, mode of transmission and interventions to prevent transmission were the focus. For child and adolescent health, top-ranked RQs were indirect effects on care, clinical status and outcomes, interventions to protect against SARS-CoV-2 infection/COVID-19 disease, and educational institute-related RQs. The cross-cutting RQs were the effects of the pandemic on availability, access, care-seeking and utilization of MNCAH services and potential solutions.

**Conclusions:**

We call on partners, including governments, non-governmental organizations, research institutes, and donors, to address this urgent research agenda.

As of June 28, 2021, the SARS-CoV-2 virus has infected more than 181 million people and resulted in more than 3.9 million deaths worldwide [1]. Compared to similarly aged adults, the case fatality rate is reported to be higher in pregnant women with SARS-CoV-2 infection or COVID-19 disease [2], and while the immediate impact on mortality is less apparent in newborns and children, severe COVID-19 disease including multisystem inflammatory syndrome temporally associated with SARS-CoV-2 infection can occur in children [3,4]. Additionally, the indirect effects of the pandemic on social, economic, educational, and other upstream determinants of health coupled with the disruption of essential maternal and child health services are likely to increase morbidity and mortality and cause significant unintended harm to the overall health and well-being of these populations [5-7].

In all countries, the COVID-19 pandemic has led to major changes to all levels of health care service delivery, and disruptions to access and use at the individual level driven by personal, social, economic, and other factors [8-11]. Most of the service delivery changes are driven by fear and the need for infection risk reduction with experimental solutions not necessarily informed by evidence. Further, little is known about the short- and long-term impact of such adaptations [12]. Additionally, there remain many gaps in understanding the short- and long-term effects of SARS-CoV-2 infection and COVID-19 disease in the pediatric population as well as during pregnancy. Therefore, knowledge gaps must be identified considering the new challenges facing women, newborns, children and adolescents, their health care providers, and the health system more widely. This could lead to research to better understand and treat infection and disease in these populations, and potential solutions to suitably address the problems created because of and shaped by this new context.

The World Health Organization (WHO) Maternal, Newborn, Child, Adolescent Health and Ageing (MCA) and Sexual and Reproductive Health and Research (SRH) departments undertook a global, rapid research prioritization exercise to identify the most urgent research questions to address the above need. The objective was to identify priority research questions related to maternal, newborn, child, and adolescent health (MNCAH) to help mitigate the direct and indirect effects of the COVID-19 pandemic. This research prioritization exercise was coordinated by the WHO on behalf of the WHO MCA-SRH COVID-19 research network (COVID-19 research network is a global network of researchers working on COVID-19 research on maternal, newborn, child and adolescent health, which has been convened and is coordinated by the department of MCA at WHO, Geneva. The purpose of the network is help with the harmonization, prioritization, use and sharing of COVID-19 research output on MNCAH), led by a WHO secretariat.

## METHODS

We used the Child Health and Nutrition Research Initiative (CHNRI) process with expedited steps to identify the priorities within a short period given the urgency posed by the pandemic [13]. There were three phases in this process: the preparatory phase (Jul-Aug 2020), the sourcing of research ideas (Aug-Sept 2020), followed by the curation of research ideas into research questions (Oct 2020), and scoring of the research questions (Nov-Dec 2020).

The preparatory phase (Jul- Aug 2020) involved defining the scope, domains, and themes for classification of the research ideas sought and criteria for scoring the research questions. This was done based on a review of the existing frameworks and CHNRI criteria by the WHO secretariat [14,15]. This was followed by a consultation with the global experts of the COVID-19 research network to finalize the same. Four domains were defined for the type of research: 1) discovery research – research emphasizing the need to invest in science and technology to understand the disease and expand the range of effective interventions; 2) development research – research with a focus on new and improved solutions or interventions; 3) delivery research – research that will help improve the coverage of existing interventions; and 4) descriptive research – epidemicological research directed at understanding the disease, burden, and the effects. Five themes were defined to classify the focus of research: pathogenesis, immune response, and immunogenicity; burden and risk factors; clinical characterization and management; modes and prevention of transmission; and indirect effects of the COVID-19 pandemic. The focus was on research questions that could be addressed over the next two years (2021-2023).

Research including prevention and treatment strategies or other interventions that are common to all populations, eg, preventive measures like physical distancing, handwashing or use of face mask, development of safe and effective vaccines (except specificities related to MNCAH), or effective treatments in general (eg, anticoagulants or antivirals), were not included in this exercise unless they addressed an aspect specific to the populations of interest. Only research ideas specific to MNCAH were included.

The second phase (Aug-Sept 2020) involved generating potential research ideas. We used the non-probability sampling technique (snowball sampling) to invite via email and an online link, a large group of experts in research, program planning and implementation, policymaking, government representatives, funding agencies, donor and international organizations, academia and civil society organizations from low- and middle-income as well as high- income settings, to submit their research ideas in the areas of maternal and newborn health, child and adolescent health or cross-cutting or health systems. The experts were requested to suggest up to three research ideas for any of the five identified themes in their self-reported field of expertise (eg, maternal and newborn health, child and adolescent health, and/or cross-cutting or health systems). The research ideas were curated by the WHO secretariat comprising technical experts in each area (ie, maternal health, newborn health, child and adolescent health, and cross-cutting-health systems; n = 9). The curation steps involved tasks such as identifying the main idea and rephrasing the research question where the idea was not clearly articulated, separating different research ideas presented together, removing duplicate ideas, combining similar research ideas, and improving the clarity of language, as required. For scoring, we categorized the research questions in the area of maternal and newborn health into two separate areas- maternal health and newborn health, respectively. In the cross-cutting or health systems area, we retained only those research questions in the list which were specific to maternal, newborn, child, and adolescent health. We excluded the generic questions, eg, health systems interventions that could affect all populations or those which were covered in individual areas. All curated questions in the discovery, development, and delivery domains were presented in the PICO (P: population, I: intervention, C: comparison/control, O: outcome) format and the PEO (P: population, E: exposure, O: outcome) format for epidemiological research (description domain), whenever possible. The secretariat ensured that the fidelity to the originally submitted research idea by the participants was maintained as much as possible in the process to develop the final list of research questions to be scored.

In the third phase (Nov-Dec 2020), the WHO secretariat contacted all the experts who were approached in the second phase for providing research ideas for scoring the final list of research questions. We shared, along with scoring instructions, the weblink to the final list of curated research questions in the four areas (maternal health, newborn health, child and adolescent health, and cross-cutting- health systems) with experts who consented to score the research questions in that area according to their self-reported field of expertise. Experts in maternal and newborn health scored both maternal health and newborn health research questions. Research questions in the discovery, development, and delivery domains were scored using all the six pre-defined criteria (**Box 1**), while those in the description domain were scored using only three of the six criteria (answerability, effectiveness, timeliness).

Box 1 **Criteria and scoring scale for scoring research questions (RQ).**

**Step 1.** Intermediate score for each criterion:

Intermediate score for criterion 1 = sum of score for Q1 for criterion 1/number of respondents who scored criterion 1 (excludes blank answer)

**Step 2.** Research priority score (RPS):

RPS for research questions in discovery, development and delivery domain = (intermediate score for criterion 1 + intermediate score for criterion 2 + intermediate score for criterion 3 + intermediate score for criterion 4 + intermediate score for criterion 5+ intermediate score for criterion 6)/ 6

RPS for research questions on description domain = (intermediate score for criterion 1 + intermediate score for criterion 2 + intermediate score for criterion 6)/ 3

**Analysis and ranking.** The research priority score (RPS) was calculated for each research question. The RPS is an average of the intermediate scores for each criterion. The intermediate score for each criterion was calculated by summing the score for each criterion and dividing by the number of respondents who scored that criterion, excluding those who scored blank. Thus, the research priority score for a research question could range from a maximum of 1 to a minimum of 0 (**Box 2**).

Box 2 **Formulas for calculation of research priority scores.**

1. **Answerability**- The RQ is likely to be clearly answered through ethical research

2. **Effectiveness**- The RQ is likely to result in effective interventions/programs to mitigate the direct and/or indirect effects of COVID-19 on MNCAH outcomes of interest

3. **Deliverability**- The RQ is likely to lead to solutions that are deliverable through existing systems within the current context of the COVID-19 pandemic

4. **Maximum potential impact**-The RQ is likely to produce results that are likely to have a large effect on MNCAH outcomes of interest within the current context of the COVID-19 pandemic

5. **Equitable**- The RQ is likely to lead to interventions/programs that will be accessible to the entire MNCA population irrespective of income or socio-economic strata or preferentially to MNCA population

6. **Timeliness**- The RQ is likely to be answered rapidly such that the results are useful to address challenges of the current COVID-19 pandemic

Scoring scale for each criterion.

1 = Yes, research question meets the criterion

0 = No, the research questions does not meet the criterion; or

Blank (no score) = Do not want to score on this criterion

The research questions were ranked based on the RPS within each area (maternal health, newborn health, child and adolescent health, and cross-cutting or health systems). The top 10 research questions with the highest RPS in maternal health, newborn health, and child and adolescent health and the top 5 research questions in the cross-cutting-health systems area were presented as the priority research questions for that area.

Furthermore, the research questions were ranked by domains and themes within each area and presented the top five research questions in each domain and theme.

Patients and public were not involved in the current study.

## RESULTS

A total 206 experts contributed to 664 research ideas in three areas- 237 on maternal and newborn health (36%), 226 on child and adolescent health (34%), and 201 for cross-cutting or health systems area (30%).

Of them approximately 51% were researchers, 22% were program managers, 7% were clinicians/academia, 6% were policy makers/government officials, 6% were research funders or donors and 7% were in multiple roles. About 46% were based in low and lower middle-income countries. More than half of the participants reported involvement in COVID-19 research, and involvement in programmatic response (38%) and developing guidelines or policy related to the COVID-19 pandemic (35%) (Table S1a in the **Online Supplementary Document**).

Following curation, research questions in the area of maternal and newborn health were separated, and 220 final research questions in four groups (maternal health, n = 31; newborn health, n = 45; child and adolescent health, n = 118; and cross-cutting or health systems, n = 26) progressed to scoring.

A total of 132 experts consented to the process (maternal and newborn health = 61, child and adolescent health = 51, and cross-cutting-health systems = 20), of whom 121 (92%) experts provided scores. The maternal questions were scored by 58 out of the 61 maternal and newborn health experts (95%), newborn questions by 56 out of the 61 maternal and newborn health experts (92%), child and adolescent health questions by 43 out of the 51 child and adolescent health experts (84%) and cross-cutting questions by all 20 cross-cutting-health systems experts (100%).

The profile of experts who scored the final research questions in each area is provided in Table S1b in the **Online Supplementary Document**.

### Research priorities in maternal health

The median score in this area was 0.83 ranging from 0.48 to 0.90 (IQR 0.75 to 0.85; n = 29). Three similar questions with the same research priority score were combined into one question, resulting in a total of 29 questions for maternal health. The questions that were combined pertained to how the COVID-19 pandemic has affected the health care-seeking behavior for antenatal care (score 0.88), childbirth care (score 0.88); and postnatal care (score 0.88).

The top ten research questions from among 29 research questions scored in the area of maternal health are listed in **Table 1****.**

**Table 1:** Top ten research priority questions on maternal health ranked by research priority score

Research priorities on maternal health	Research priority score
1. How can the access to postnatal including home-based health services affected by the pandemic be improved (eg, using telemedicine)?	0.90
2. In pregnant women with SARS-CoV-2 infection or COVID-19 disease, what are the risk (eg, co-morbidities, co-infection) and determinants (eg, malnutrition, body composition) of adverse maternal and perinatal outcomes (eg, maternal morbidity, stillbirth, etc.)?	0.89
3. How has the COVID-19 pandemic affected health-seeking behavior for antenatal care, childbirth, or postnatal care (eg, frequency of visits, birth at health facilities, family planning advice, etc.)?	0.88
4. How can the access to maternal and perinatal health services affected by the pandemic be improved? (eg, skilled delivery, telemedicine, use of point of care ultrasound)	0.87
5. Is the safety and efficacy of the SARS-CoV-2 vaccine different in pregnant women compared to non-pregnant women?	0.87
6. What are the effective drugs/procedures to manage women with COVID-19 disease in the 1st, 2^nd,^ and 3rd trimester of pregnancy?	0.87
7. How can the provision of essential maternal and perinatal health commodities affected by the pandemic be improved?	0.86
8. In postpartum women with COVID-19, what are the risk and determinants of transmission of SARS-CoV-2 to the newborn by breastfeeding or breastmilk?	0.85
9. Are pregnant women with complications such as pregnancy-induced hypertension, pre-eclampsia, gestational diabetes, malnutrition, anemia, HIV-1, and malaria at increased risk of SARS-CoV-2 infection or COVID-19 disease compared to women with uncomplicated pregnancies?	0.85
10. What are the risk and determinants of severe disease or death in pregnant women with SARS-CoV-2 infection or COVID-19 disease (eg, sociodemographic, immunological/hormonal, microbiota, nutritional)?	0.85

### Newborn health

The median score in this area was 0.77 ranging from 0.44 to 0.90 (IQR 0.70 to 0.82; n = 45). The top ten research questions from among the 45 research questions scored in the area of newborn health are listed in Table 2.

**Table 2:** Top ten research priority questions on newborn health ranked by research priority score

Research priorities on newborn health	Research priority score
1. What are the most effective treatment strategies for symptomatic newborns with SARS-CoV-2 infection, especially those with respiratory illness?	0.90
2. What is the effectiveness of different interventions (eg, maternal hygiene, facemask use, maternal-newborn separation, etc.) to prevent neonatal acquisition of SARS-CoV-2 in the early postnatal period in the hospital and at home while breastfeeding, during skin-to-skin contact or kangaroo mother care, especially if the mother has symptomatic COVID-19 disease?	0.87
3. Is SARS-CoV-2 transmissible to healthy infants including newborns of mothers infected with the virus? What are the routes of transmission during in-utero, intrapartum, and postnatal periods (eg, transplacental, contact with virus present in vaginal or fecal secretions during delivery, breastmilk, etc.)? Does the incidence and severity of infection in the newborns differ by the timing, transmission, or mode of delivery (eg, cesarean vs vaginal delivery)?	0.87
4. What are the clinical presentations of SARS-CoV-2 infection in newborns? What is the burden of severe COVID-19 disease in newborns?	0.87
5. What proportion of newborns infected with SARS-CoV-2 presenting for care with hypoxemia receive oxygen therapy, and what is the case fatality rate in this population?	0.86
6. What is the most effective way to prevent the transmission of SARS-CoV-2 to preterm newborns receiving kangaroo mother care?	0.86
7. What are the major risk and/or protective factors (eg, breastfeeding, skin-to-skin care or kangaroo mother care, low birth weight, Bacillus Calmette Guerin or other existing vaccinations, HIV-1 exposure, etc.) for the acquisition of SARS-CoV-2 infection or development of COVID-19 disease or severe disease in newborns of SARS-CoV-2 infected mothers?	0.85
8. In newborns with severe COVID-19 disease, is the addition of dexamethasone to the standard of care more effective in improving newborn survival compared to the standard of care alone?	0.85
9. Which is the most appropriate diagnostic sample for diagnosis of SARS-CoV-2 infection in newborns (eg, oral secretion, nasal swab, fecal swab, cord or peripheral blood, etc.?	0.84
10. What is the impact of maternal SARS-CoV-2 infection on newborn health outcomes (eg, intrauterine growth restriction, prematurity, and birth asphyxia), and how can this be mitigated?	0.83

### Child adolescent health

The median score in this area was 0.79 ranging from 0.47 to 0.95 (IQR 0.70 to 0.82; n = 111).

The top ten research questions from among the 111 research questions scored in the area of child and adolescent health are listed in **Table 3**.

**Table 3:** Top ten research priority questions on child and adolescent health ranked by research priority score

Research priorities on child and adolescent health	Research priority scores
1. How can routine vaccination services for children be sustained/improved during the COVID-19 pandemic?	0.95
2. What are the effective and safe strategies for in-class education during the COVID-19 pandemic? What is the attack rate within schools, and what interventions (eg, face masks, physical distancing, hand disinfection/washing, etc.) are effective for prevention of transmission of SARS-CoV-2 in schools, by age/school year and by type of school (eg, day vs boarding)?	0.94
3. What are the most effective strategies for communication about the prevention of SARS-CoV-2 infection to adolescents and young adults?	0.93
4. What are the protective factors, including breastfeeding/feeding practices, maternal vaccination against severe COVID-19 disease in infants, children, and adolescents (0-19 y), and do these differ by age?	0.92
5. What is the impact of comorbidities (eg, asthma, diabetes, undernutrition, overweight or obesity, HIV-1 exposure or HIV-1 infection, etc.) in children and adolescents (0-19 y) with SARS-CoV-2 infection on development of disease and on its clinical severity and outcome?	0.91
6. What is the effect of reopening schools on SARS-CoV-2 transmission among different age groups in the general population (based on real-life data)?	0.91
7. What is the sensitivity and specificity of SARS-CoV-2 RT-PCR testing in children?	0.90
8. What is the impact of the COVID-19 pandemic on essential child health services (both at a community level and facility level)?	0.90
9. What are the best risk-stratification tools (eg, Integrated Management of Childhood Illness algorithm, biomarkers, clinical scores, etc.) to triage sick children with suspected SARS-CoV-2 infection or COVID-19 disease, and with sufficiently good performance to predict the severity and outcome?	0.88
10. What is the prevalence of SARS-CoV-2 infection in breastfed vs non-breastfed infants?	0.88

### Cross-cutting/Health systems

The median score in this area was 0.83 ranging from 0.50 to 0.93 (IQR 0.73 to 0.86; n = 25). Two interlinked questions ranked serially with a score of 0.93 and 0.92 were combined into one question, resulting in a total of 25 questions for cross-cutting or health systems area. The top five questions from among the 25 research questions scored in the cross-cutting or health systems area are listed in **Table 4**.

**Table 4:** Top five research priority questions in cross-cutting or health systems area, ranked by research priority score

Research priorities in cross-cutting/health system domain	Research priority score
1. What are safe and cost-effective approaches to provide oxygen to pregnant women, newborns, and children who need oxygen in low- and middle-income countries with limited oxygen supply?	0.93
2. What are the barriers to the provision of essential reproductive, maternal, newborn, child, and adolescent health services during the COVID-19 pandemic? What are proven health system approaches/solutions that countries have adapted or implemented to ensure continuity of essential reproductive, maternal, newborn, child, and adolescent health services during the COVID-19 pandemic?	0.93
3. What are the effective health system adaptations to sustain care-seeking and support or resume high-quality essential services for women, newborns, children, and adolescents in COVID-19 and post-COVID-19 contexts?	0.91
4. To what extent are resources (health workers, oxygen support, infection, and prevention control supplies) being diverted from women, newborns, and children or routine services to provide care for COVID-19 disease?	0.90
5. What different models have countries taken to adapt or modify health services for maternal and newborn health during the COVID-19 pandemic, and what is the effect of the new adapted or modified or redesigned COVID-19 models of care (eg, remote antenatal and postnatal services replacing in-person contact) on access, coverage, quality of care and outcomes of mothers and newborns, especially for marginalized populations?	0.88

The ranking of the questions by theme and domain is presented in Tables S2a and S2b in the **Online Supplementary Document****.**

Median (IQR) of the RPS across different areas remained similar irrespective of income classification of the scorer’s country of work or residence (high, upper-middle, lower-middle, and lower-income) (Table S3 in the **Online Supplementary Document**). The ranking of the top ten research priorities by income classification of the scorer’s country of work is presented for each area (Tables S4a, S4b, S4c, and S4d in the **Online Supplementary Document**).

## DISCUSSION

In March 2020, the WHO published a Global Research Roadmap to target and respond to SARS-CoV-2 with immediate, mid-term, and longer-term priorities to build a robust global research response based on the deliberations during the Global Research Forum, and other global organizations have made similar calls [14,152]. The document captured major thematic areas (from virus natural history to social sciences in the outbreak response) and strategic actions, but it was beyond the scope to capture the unique aspects of research related to the health and well-being specific to mothers, newborns, children, and adolescents. This study was conducted with a focus on the maternal, newborn, child, and adolescent populations, to identify priority research needs that may help in addressing the direct and indirect effects of the COVID-19 pandemic and mitigation interventions on these populations.

In maternal health, three key focus areas emerged: 1) the indirect effects of the COVID-19 pandemic on care for women (including health-seeking behavior, access to care, and provision of care) and measures to improve access and provision of care, 2) the health risks and outcomes for pregnant women in the context of SARS-CoV-2 infection or COVID-19 disease including perinatal outcomes (stillbirth, fetal growth restriction); and 3) prevention and management of SARS-CoV-2 infection or COVID-19 disease during pregnancy including the safety and efficacy of SARS-CoV-2 vaccine for pregnant women.

In newborn health, the focus was on clinical characterization and management of SARS-CoV-2 infection or COVID-19 disease (most effective treatment strategies, clinical presentations, burden of severe disease, risk and protective factors especially breastfeeding, kangaroo mother care, Bacillus Calmette Guerin or other vaccinations, most yielding sample for diagnosis, etc.), mode of possible transmission from mother to child (routes of transmission, incidence, and severity by route of transmission), and effectiveness of different interventions to prevent transmission from an infected and/or symptomatic mother.

For child and adolescent health, there were four focus areas: 1) Indirect effect of COVID-19 pandemic on care (impact on essential health services and maintaining routine vaccination services); 2) Clinical status and outcomes of SARS-CoV-2 infection or COVID-19 disease in this population (impact of comorbidities on clinical severity of infection, risk stratification tools to predict severity and outcome of infection); 3) Interventions that might protect against SARS-CoV-2 infection or COVID-19 disease; and 4) Educational institutes (effective and safe strategies/intervention for in-class education and effect of reopening of schools on SARS-CoV-2 transmission). The cross-cutting priorities focused on: 1) safe and effective access to oxygen therapy; 2) effects of the COVID-19 pandemic on availability, access, care-seeking, and utilization of MCH services; and 3) potential solutions.

These global priorities provide a starting point for countries to tailor these research questions to their local realities and priorities, with a note that the research on these priorities should not be separated from broader research priorities in the area of maternal, newborn, child, and adolescent health. Learning across different contexts will help inform how different interventions might be adapted or replicated to answer some of the priority research questions. Sharing evidence generated by research and lessons from the implementation of the identified solutions will be crucial for ensuring effective and efficient responses.

The current priorities have been developed using adapted CHNRI methodology in a transparent, ethical, and equitable manner using a systematic process [16], and highlight key gaps in knowledge and are relevant both for clinical practice and programmatic implementation. There have been other research prioritization exercises in the context of the COVID-19 pandemic which are broader in scope [17], or specific to a particular area, eg, surgery or mental health [18,19], and one very recent one on maternal and newborn health from the African continent [20], but we are not aware of any other attempt at defining priorities across the area of maternal, newborn, child and adolescent health at a global level. The added value of the current study lies in the engagement of a large number of global experts for generating ideas and scoring the research questions.

Our exercise highlights the importance of generating evidence to understand effective strategies and intervention measures to mitigate the direct and indirect effects of the COVID-19 pandemic on mothers, newborns, children, and adolescents. Research on some of the identified priorities is already under way, eg, epidemiology of COVID-19 disease in pregnancy to determine risk factors and associations with adverse maternal and neonatal outcomes [21], maternal to newborn transmission [22], viral transmission following implementation of COVID-19 preventive measures in schools [23-25] and the effect of reopening schools on SARS-CoV-2 transmission [26,27], etc., which support the validity of the focus on these priorities in the current exercise. We hope that the current exercise will bring to focus issues that have not yet been addressed and appropriate research in these areas. This could help us be prepared with evidence-based approaches to minimizing the adverse impact on mothers, newborns, children, and adolescents during the future waves of the pandemic.

The ranking of the top ten research priorities was similar irrespective of income classification of the scorer’s country of work except for a few questions in each area, eg, the question on the safety and efficacy of the SARS-CoV-2 vaccine in pregnant women was ranked higher by scorers working in high and upper-middle income countries, while the one on documenting the impact of the COVID-19 pandemic on essential child health services at the community and facility levels was ranked higher by scorers working in low and lower-middle income countries. This reflects that the priorities are influenced by the context and may differ across settings.

One limitation of this study pertains to the fact that the pandemic is a fast-moving field, wherein new emerging issues arise quickly while the exercise is in progress, and some issues such as the recent circulation of new viral variants safety and efficacy of SARS-CoV-2 vaccines for children and adolescents are not adequately captured. Another limitation could be that the self-selected experts may have their interests for priority research areas. However, in this exercise, we tried to approach as many experts as possible to participate to generate research ideas and to score the research questions. Therefore, we think there is limited scope for selection bias in the process. It is also acknowledged that while the pandemic is not yet over, with many countries experiencing a second or anticipating further waves, there is still a need to initiate post-pandemic planning, which could be informed by identified research priorities.

## CONCLUSION

Research in the area of SARS-CoV-2 infection and COVID-19 disease has been progressing substantially since the beginning of the pandemic, driven by experts across the globe. However, key issues like those identified in this study present collective knowledge and experience of a large, diverse group of global experts which was a challenge but a timely achievement. The identified priorities now need careful planning, coordination, and quick implementation to be effective. We call on partners, including governments, non-governmental organizations, research institutes, and donors, to collaborate to ensure that this urgent research agenda be addressed with a specific focus on priority areas that have not yet been taken up for research.

## Additional material


Online Supplementary Document
