# Supplementary material for: Global research priorities on COVID-19 for maternal, newborn, child and adolescent health
Source: J Glob Health. 2021 Nov 20;11:04071. doi: 10.7189/jogh.11.04071 (PMC8645217; doi:10.7189/jogh.11.04071)
Supplement: Online Supplementary Document [file jogh-11-04071-s001.pdf]

Title. A global research prioritization exercise on COVID-19 for maternal, newborn, child and adolescent health

ONLINE SUPPLEMENTARY DOCUMENT

Table 1a. Profile of experts who contributed the research ideas

	**N=206 (100%)**
**Area/Domain of work***	
Maternal health	74 (35.9%)
Newborn health	97 (47.1%)
Child health	118 (57.3%)
Adolescent health	51 (24.6%)
Other	34 (16.5%)
**Predominant role in the area of work**	
Researcher	105 (51.0%)
Program planning and implementation	46 (22.3%)
Research funder or donor	12 (5.8%)
Policy maker/Government	12 (5.8%)
Clinicians/Academia	13 (6.3%)
Multiple roles	9 (4.4%)
Other	9 (4.4%)
**Setting**	
Upper income	78 (37.9%)
Upper middle	33 (16.0%)
Lower middle	56 (27.9%)
Lower income	36 (17.5%)
**Current role in the context of COVID-19 pandemic***	
Managing COVID-19 patients	32 (15.5%)
Involved in COVID-19 related research	116 (56.3%)
Involved in programmatic response to COVID-19	79 (38.3%)
Developing guidelines or policy related to the COVID-19 pandemic	73 (35.4%)
Providing financial support to COVID related research or programmatic response	14 (6.8%)
Other	9 (4.4%)

**Due to multiple choice option, the total number does not tally 206.*

Table 1b. Profile of experts who scored research questions in different areas

	Maternal health	Newborn health	Child and Adolescent health	Cross-cutting
**Total no. of scorers**	**N=58 (100%)**	**N=56 (100%)**	**N=43 (100%)**	**N=20 (100%)**
**Gender**				
Male	27 (47%)	24 (43%)	25 (58%)	11 (55%)
Female	31 (53%)	32 (57%)	18 (42%)	9 (45%)
**Predominant role in the area of work**				
Academia/research	47 (81%)	43 (77%)	34 (79%)	9 (45%)
Government: Policy, program planning, and management	5 (9%)	5 (9%)	0	0
Non-Government: Policy, program planning, and management	6 (10%)	8 (14%)	9 (21%)	11 (55%)
**Setting (by country of residence)**				
Upper and upper middle	45 (78%)	45 (80%)	32 (74%)	11 (55%)
Lower and lower middle	13 (22%)	11 (20%)	11 (26%)	9 (45%)
**Setting (by country of work)**				
Upper and upper middle	24 (41%)	22 (39%)	14 (33%)	5 (25%)
Lower and lower middle	32 (55%)	31 (55%)	25 (58%)	13 (65%)
Work at global level	2 (3%)	3 (5%)	4 (9%)	2 (10%)

Ranking of final research questions by domain and theme

New questions, the ones that didn't feature in the top ten research priorities by area are marked in **blue.**

Table 2a. Top ranked research questions by domain (stratified by area; top 5 in each area)

Domain	Research priority questions
Discovery (new interventions)	**Maternal health**<br>1. Is the safety and efficacy of the Severe Acute Respiratory Syndrome Coronavirus 2 (SARS-CoV-2) vaccine different in pregnant women compared to non-pregnant women?<br>2. What are the effective drugs or procedures to manage women with COVID-19 disease in the 1st, $2^{nd,}$ and 3rd trimester of pregnancy?<br>3. What are the best laboratory or clinical markers for severe COVID-19 disease in pregnant women?<br><br>**Newborn health**<br>1. In newborns with severe COVID-19 disease, is the addition of dexamethasone to the standard of care more effective in improving newborn survival compared to the standard of care alone?<br>2. Can Bacillus Calmette Guerin (BCG) vaccination at birth modify the immune response in infants who receive the SARS-CoV-2 vaccine once it becomes available for this age group?<br>3. What are the mechanisms by which breastfeeding can prevent SARS-CoV-2 infection or the development of COVID-19 disease or severe disease (e.g., the presence of SARS-CoV-2 antibody in human milk) in the newborn?<br>4. What are the incidence and the mechanism of the late-onset (onset after 48 hours of life) COVID-19 disease in newborns?<br>5. Do infants mount an immunological response to SARS-CoV-2?<br><br>**Child and adolescent health**<br>1. Is there a non-invasive test to detect SARS-CoV-2 that is accurate and quick to use with children?<br>2. What is the best point of care SARS-CoV-2 RT-PCR assay in children?
Development (Improved intervention)	**Maternal health**<br>1. How can transmission between a SARS-CoV-2 infected parent to their newborn be prevented?<br><br>**Newborn health**<br>1 What are safe and cost-effective approaches to provide oxygen to pregnant women, newborns, and children who need oxygen in resource-poor countries with limited oxygen supply?<br>2 What is the effectiveness of different interventions (e.g., maternal hygiene, use of facemask, maternal-newborn separation, etc.) to prevent neonatal acquisition of SARS-CoV-2 in the early postnatal period in the hospital and at home while breastfeeding, skin-to-skin contact, and kangaroo mother care (KMC), especially if the mother has symptomatic COVID-19 disease?

	**Child and adolescent health**<br>1 What are the effective and safe strategies for in-class education during the COVID-19 pandemic? What is the attack rate within schools and what interventions (e.g. face masks, physical distancing, hand disinfection/washing, etc.) are effective for the prevention of transmission of SARS-CoV-2 in schools, by age/school year and by type of school (e.g. day vs boarding)?<br>2 What are the most effective strategies for communication about the prevention of SARS-CoV-2 infection to adolescents and young adults?<br>3 What are the best risk-stratification tools (e.g., Integrated Management of Childhood Illness algorithm, biomarkers, clinical scores, etc.) to triage sick children with suspected SARS-CoV-2 infection or COVID-19 disease, and with sufficiently good performance to predict the severity and outcome?<br>4 Among children with COVID-19 disease and severe respiratory distress, does early use of CPAP compared to no CPAP, reduce the subsequent use of invasive mechanical ventilation?<br><br>**Cross-cutting**<br>1 How can the spread of SARS-CoV-2 infection be controlled and the adverse impact of COVID-19 disease be mitigated in children and families who are displaced and living in fragile settings?<br>2 Which innovations are effective in reducing the unintended adverse consequences of service reorganization during the COVID-19 pandemic on optimizing positive pregnancy, childbirth, and postnatal experiences and ensuring safe outcomes for childbearing women and their neonates?
Delivery (Delivery of existing interventions)	**Maternal health**<br>1. How can the access to postnatal including home-based health services affected by the pandemic be improved (e.g., using telemedicine)?<br>2. How can the access to maternal and perinatal health services affected by the pandemic be improved? (e.g., skilled delivery, telemedicine, use of point of care ultrasound)<br>3. How can the provision of essential maternal and perinatal health commodities affected by the pandemic be improved?<br>4. Which package of personal protective equipment for mothers, companions, and health providers is associated with the lowest risk of SARS-CoV-2 transmission in the delivery room?<br>5. Which mode of delivery (vaginal or cesarean section) is associated with the best clinical outcomes in pregnant mothers with severe COVID-19 disease?

	**Newborn health**<br>1. What are the most effective treatment strategies for symptomatic newborns with SARS-CoV-2 infection, especially those with respiratory illness?<br>2. What is the most effective way to prevent the transmission of SARS-CoV-2 to preterm newborns receiving kangaroo mother care?<br><br>**Child and adolescent health**<br>1. How can routine vaccination services for children be sustained/improved during the COVID-19 pandemic?<br>2. How can schools be utilized as platforms to deliver equitable academic and mental health interventions to children and adolescents 0-19 years of age during and post the COVID-19 pandemic?<br>3. What is the optimal treatment for multisystem inflammatory syndrome in children temporally associated with SARS-CoV-2 infection (MISC)?<br>4. What are the effective interventions to prevent malnutrition in children during the COVID-19 pandemic lockdown?<br>5. How can technology assist with the delivery of evidence-based psychosocial interventions to prevent or treat mental health problems in adolescents in the post-COVID-19 pandemic context?<br><br>**Cross-cutting**<br>1. What are the barriers to the provision of essential reproductive, maternal, newborn, child, and adolescent health services during the COVID-19 pandemic? What are proven health system approaches/solutions that countries have adapted or implemented to ensure continuity of essential reproductive, maternal, newborn, child, and adolescent health services during the COVID-19 pandemic?<br>2. What are the effective health system adaptations to sustain care-seeking and support or resume high-quality essential services for women, newborns, and children in COVID-19 and post-COVID-19 contexts?<br>3. How can we organize health services during the COVID-19 pandemic to ensure the continuation of regular maternal, newborn, child, and adolescent health care?
Descriptive (Epidemiological research)	**Maternal health**<br>1 In pregnant women with SARS-CoV-2 infection or COVID-19 disease, what are the risk (e.g., co-morbidities, co-infection) and determinants (e.g., malnutrition, body composition) of adverse maternal and perinatal outcomes (e.g., maternal morbidity, stillbirth, etc.)?

	2 How has the COVID-19 pandemic affected health-seeking behavior for antenatal care, childbirth, or postnatal care (e.g. frequency of visits, birth at health facilities, family planning advice, etc.)?<br><br>**Newborn health**<br>1 Is SARS-CoV-2 transmissible to healthy infants including newborns of mothers infected with the virus? What are the routes of transmission during the in-utero, intrapartum, and the postnatal periods (e.g., transplacental, contact with virus present in vaginal or fecal secretions during delivery, breastmilk, etc.)? Does the incidence and severity of infection in the newborn differ by the timing or transmission or mode of delivery (e.g., cesarean vs. vaginal delivery)?<br>2 What are the clinical presentations of SARS-CoV-2 infection in newborns? What is the burden of severe COVID-19 disease in newborns?<br>3 What proportion of newborns infected with SARS-CoV-2 presenting for care with hypoxemia receive oxygen therapy, and what is the case fatality rate in this population?<br><br>**Child and adolescent health**<br>1 What are the protective factors including breastfeeding/feeding practices, maternal vaccination, against severe COVID-19 disease in infants, children, and adolescents (0-19 years), and do these differ by age?<br>2 What is the impact of comorbidities (e.g., asthma, diabetes, undernutrition, overweight or obesity, <u>HIV-1 exposure or HIV-1 infection, etc.</u>) in children and adolescents (0-19 years) with SARS-CoV-2 infection on development of disease and its clinical severity and outcome?<br>3 What is the effect of reopening schools on SARS-CoV-2 transmission among different age groups in the general population (based on real-life data)?<br>4 What is the sensitivity and specificity of SARS-CoV-2 RT-PCR testing in children?<br>5 What is the impact of the COVID-19 pandemic on essential child health services (both at a community level and facility level)?<br><br>**Cross-cutting**<br>1. To what extent are resources (health workers, oxygen support, IPC supplies) being diverted from women, newborns, and children or routine services to provide care of COVID-19 disease?<br>2. What different models have countries taken to adapt or modify health services for maternal and newborn health during the COVID-19 pandemic and what is the effect of the new adapted or modified or redesigned COVID-19 models of care (e.g. remote antenatal and

	postnatal services replacing in-person contact) on access, coverage, quality of care and outcomes of mothers and newborns, especially for marginalized populations?<br>3. How do various factors (e.g., age, wealth or income or socioeconomic status, race or ethnicity, migrant or refugee status, etc.) influence individuals' access to COVID-19 related healthcare services (e.g., test, treatment, etc.)?<br>4. What are the effects of the COVID-19 pandemic on availability, access, care-seeking, and utilization of maternal and child health services?<br>5. How has care-seeking and quality of care (at every health service level) been affected by the COVID pandemic, and what are the features of positive outliers (i.e. settings and services that have been able to successfully maintain essential services against the odds)?

Table 2b. Top ranked research questions by theme (stratified by area; top 5 in each area)

Theme	Research priority questions
Pathogenesis, immune response, immunogenicity	**Maternal health**<br>1. What is the extent of in-utero transfer of SARS-CoV2 antibodies from infected mother to the fetus? How long after birth do the placentally transferred SARS-CoV-2 antibodies persist in the newborn baby?<br>2. In pregnant women infected with SARS-CoV-2, what are the SARS-CoV-2 immune response and longevity of the immune response to the virus?<br><br>**Newborn health**<br>1. Can BCG vaccination at birth modify the immune response in infants who receive the COVID-19 vaccine once it becomes available for use in this age group?<br>2. What are the mechanisms by which breastfeeding can prevent SARS-CoV-2 infection or the development of COVID-19 disease or severe disease in the newborn (e.g., the presence of SARS CoV 2 antibody in human milk)?<br><br>**Child and adolescent health**<br>1. Is there a non-invasive test to detect the SARS-CoV-2 virus that is accurate and quick to use with children?<br>2. After exposure to SARS-CoV-2 infection, does a child develop a protective immune response? If yes, for how long?<br>3. What is the seroprevalence of SARS-CoV-2 infection in children (0-19 years) and its comparison with other population groups by each country?

	4. What is the best point of care test SARS-CoV-2 RT-PCR assay in children?<br>5. How do premorbid HIV, TB, or malnutrition impact the immune response of infants/young children infected with SARS-CoV2?
Burden and risk factors	**Maternal health**<br>1. In pregnant women with SARS-CoV-2 infection or COVID-19 disease, what are the risk (e.g., co-morbidities, co-infection) and determinants (e.g., malnutrition, body composition) of adverse maternal and perinatal outcomes (e.g., maternal morbidity, stillbirth, etc.)?<br>2. Are pregnant women with complications such as pregnancy-induced hypertension, pre-eclampsia, gestational diabetes, malnutrition, anemia, HIV-1, malaria at increased risk of SARS-CoV-2 infection compared to women with uncomplicated pregnancies?<br>3. Are pregnant women at increased risk of SARS-CoV-2 infection or COVID-19 disease or differ in the clinical syndrome compared to women who are not pregnant?<br><br>**Newborn health**<br>1. Do SARS-CoV-2 viral load and severity of COVID-19 disease in infected pregnant women predict the acquisition of infection and development and severity of COVID-19 disease in the newborn?<br>2. Does co-infection of the mother with other infections like HIV, hepatitis, malaria, syphilis, etc. along with SARS-CoV-2 increase the risk of the newborn acquiring SARS-CoV-2 infection and developing COVID-19 disease or severe disease?<br><br>**Child and adolescent health**<br>1. What are the protective factors including breastfeeding/feeding practices, maternal vaccination against severe COVID-19 disease in infants, children, and adolescents (0-19 years), and do these differ by age?<br>**2.** What is the prevalence of SARS-CoV-2 infection in breastfed vs non-breastfed infants?<br>3. What are the risk factors (biomarkers, environmental, nutritional, social, demographic, etc.) for SARS-CoV-2 infection and COVID-19 disease in children and adolescents (0-19 years), and do these differ by age?<br>**4.** What is the impact of the COVID-19 pandemic on immunization preventable diseases (e.g. measles, pneumococcal, H. influenza, pertussis, tetanus, etc.) in children 0-19 years of age?
Clinical characterization	**Maternal health**

and management	1. What are the effective drugs/procedures to manage COVID-19 disease in the 1st, 2nd, and 3rd trimester of pregnancy?<br>2. Which mode of delivery (vaginal or cesarean section) is associated with the best clinical outcomes in pregnant mothers with severe COVID-19 infection?<br>3. Which maternal position during birth (e.g., prone with support, left lateral, other) is associated with best clinical outcomes in pregnant mothers with severe COVID-19 disease?<br><br>**Newborn health**<br>1. What are the most effective treatment strategies for symptomatic newborns with SARS-CoV-2 infection, especially those with respiratory illness?<br>2. What is the effectiveness of different interventions (e.g., maternal hygiene, use of facemask, maternal-newborn separation, etc.) to prevent neonatal acquisition of SARS-CoV-2 in the early postnatal period in the hospital and at home while breastfeeding, during skin-to-skin contact and KMC, especially if the mother has symptomatic COVID-19 disease?<br>3. What are the clinical presentations of SARS-CoV-2 infection in newborns? What is the burden of severe COVID-19 disease in newborns?<br>4. What proportion of newborns infected with SARS-CoV-2 presenting for care with hypoxemia receive oxygen therapy, and what is the case fatality rate in this population?<br><br>**Child and adolescent health**<br>1. What is the impact of comorbidities (e.g., asthma, diabetes, undernutrition, overweight or obesity, HIV-1 exposure or HIV-1 infection, etc.) in children and adolescents (0-19 years) with SARS-CoV-2 infection or COVID-19 disease on the clinical severity of the infection and outcome?<br>2. What is the sensitivity and specificity of SARS-CoV-2 RT-PCR testing in children?<br>3. What are the best risk-stratification tools (IMCI algorithm, biomarkers, clinical scores, etc.) to triage sick children with COVID-19 disease, and with sufficiently good performance to predict the severity and outcome?<br>4. What combinations of signs and symptoms (as used in iCCM/IMCI) best predict COVID-19 in children presenting with illness to a primary health care facility?<br>5. Among children with COVID-19 disease and severe respiratory distress, does early use of CPAP compared to no CPAP, reduce the subsequent use of invasive mechanical ventilation?

Modes of transmission and prevention of transmission	**Maternal health**<br>1. In postpartum women infected with SARS-CoV-2, what are the risk and determinants of SARS-CoV-2 transmission to the newborn by breastfeeding or breastmilk?<br>2. How can transmission between a SARS-CoV-2 infected parent to their newborn be prevented?<br>3. In SARS-CoV-2 infected pregnant women, what is the risk and determinants of viral transmission to the fetus and newborn during pregnancy and childbirth?<br>4. What proportion of lactating mothers infected with SARS-CoV-2 secrete replicating live virus in their breastmilk? What is the risk of transmission of SARS-CoV-2 to the newborn if fed breastmilk containing the live replicating virus?<br>**Newborn health**<br>1. Is SARS CoV-2 transmissible to healthy infants including newborns of mothers infected with the virus? What are the routes of transmission during the in utero, intrapartum and postnatal periods (e.g., transplacental, contact with virus present in vaginal or fecal secretions during delivery, breastmilk, etc.)? Does the incidence and severity of infection in the newborn differ timing of transmission or mode of delivery (e.g., cesarean vs vaginal delivery)?<br><br>**Child and adolescent health**<br>1. What are the effective and safe strategies for in-class education during the COVID-19 pandemic? What is the attack rate within schools and what interventions (e.g. face masks, physical distancing, hand disinfection/washing, etc.) are effective for the prevention of transmission of SARS-CoV-2 in schools, by age/school year and by type of school (e.g. day vs boarding)?<br>2. What are the most effective strategies for communication about the prevention of SARS-CoV-2 to adolescents and young adults?<br>3. What is the effect of reopening schools on SARS-CoV-2 transmission among different age groups in the general population (based on real-life data)?<br>4. What is the rate of transmission of SARS-CoV2 among children and adolescents 0-19 years of age, overall and by age?
Indirect effects of the pandemic	**Maternal health**<br>1. How can the access to postnatal including home-based health services affected by the pandemic be improved (e.g., using telemedicine)?<br>2. How has the COVID-19 pandemic affected health-seeking behavior for antenatal care, childbirth, or postnatal care (e.g. frequency of visits, birth at health facilities, family planning advice, etc.)?Is the

	safety and efficacy of the SARS-CoV-2 vaccine different in pregnant women compared to non-pregnant women?<br>3. How can the access to maternal and perinatal health services affected by the pandemic be improved (e.g. skilled delivery, telemedicine, use of point of care ultrasound)?<br>4. How can the provision of essential maternal and perinatal health commodities affected by the pandemic be improved?<br><br>**Newborn health**<br>1. What is the effect of the COVID-19 pandemic on care-seeking for newborn illness? What are the major concerns and barriers faced by families in seeking care at health facilities for a sick newborn during the pandemic?<br>2. What is the best screening test for SARS-CoV-2 for newborns born to women who have tested positive for SARS-CoV-2 on RT-PCR?<br>3. What is the impact of the COVID-19 pandemic on the emergency admission rates for sick newborns and infants?<br>4. What is the impact of enablers and inhibitors of adoption of digital health solutions on antenatal and postnatal care in LMIC settings during the COVID-19 pandemic?<br><br>**Child and adolescent health**<br>1. How can routine vaccination services for children be sustained/improved during the COVID-19 pandemic?<br>2. What are the effective and safe strategies for in-class education during the COVID-19 pandemic? What is the attack rate within schools and what interventions (e.g. face masks, physical distancing, hand disinfection/washing, etc.) are effective for the prevention of transmission of SARS-CoV-2 in schools, by age/school year and by type of school (e.g. day vs boarding)?<br>**3.** What is the impact of the COVID-19 pandemic on essential child health services (both at a community level and facility level)?<br><br>**Cross-cutting**<br>1. What are safe and cost-effective approaches to provide oxygen to pregnant women, newborns, and children who need oxygen in resource-poor countries with limited oxygen supply?<br>2. What are the barriers to the provision of essential RMNCAH services during the COVID-19 pandemic? What are proven health system approaches/solutions that countries have adapted or implemented to ensure continuity of essential MNCAH services during the COVID-19 pandemic?<br>3. What are the effective health system adaptations to sustain care-seeking and support or resume high-quality essential services for women, newborns, and children in COVID-19 and post-COVID-19 contexts?

	4. To what extent are resources (health workers, oxygen support, IPC supplies) being diverted from women, newborns, and children / routine services to provide care of COVID-19 disease?<br>5. What different models have countries taken to adapt/modify health services for maternal and newborn health during the COVID-19 pandemic and what is the effect of the new adapted/modified/redesigned COVID-19 models of care (e.g. remote antenatal and postnatal services replacing in-person contact) on access, coverage, quality of care and outcomes of mothers and newborns, especially for marginalized populations?

Table 3. Research priority Score (RPS) for all research questions in the four areas by income classification of scorer's country of work and country of residence

	**By country of work**		**By country of residence**	
	RPS score for scorers from upper and upper middle-income countries; Median (IQR)	RPS score for scorers from low and lower middle-income countries; Median (IQR)	RPS score for scorers from upper and upper middle-income countries; Median (IQR);	RPS score for scorers from low and lower middle-income countries; Median (IQR)
Maternal health	0.85 (0.79 to 0.91); n=24	0.80 (0.71 to 0.85); n=32	0.83 (0.75 to 0.88); n=45	0.79 (0.73 to 0.83); n=13
Newborn health	0.80 (0.76 to 0.89); n=22	0.73 (0.68 to 0.79); n=31	0.79 (0.71 to 0.85); n=45	0.75 (0.67 to 0.81); n=11
Child and Adolescent health	0.76 (0.68 to 0.83); n=14	0.80 (0.72 to 0.86); n=25	0.77 (0.70 to 0.82); n=32	0.82 (0.73 to 0.89); n=11
Cross-cutting	0.79 (0.66 to 0.89); n=5	0.84 (0.72 to 0.88); n=13	0.79 (0.69 to 0.85); n=11	0.85 (0.76 to 0.89); n=9

Table 4a. Ranking of the top ten research questions on maternal health by income classification of the scorer's country of work

Overall ranking of top ten research questions on maternal health		Ranking by World Bank country income classification	
		Upper and upper middle-income countries	Lower and lower-middle income countries
1	How can the access to postnatal including home-based health services affected by the pandemic be improved (e.g., using telemedicine)?	2*	1*
2	In pregnant women with SARS-CoV-2 infection or COVID-19 disease, what are the risk (e.g., co-morbidities, co-infection) and determinants (e.g., malnutrition, body composition) of adverse maternal and perinatal outcomes (e.g., maternal morbidity, stillbirth, etc.)?	5*	2
3	How has the COVID-19 pandemic affected health-seeking behavior for antenatal care, childbirth, or postnatal care (e.g. frequency of visits, birth at health facilities, family planning advice, etc.)?	3	3

4	How can the access to maternal and perinatal health services affected by the pandemic be improved? (e.g. skilled delivery, telemedicine, use of point of care ultrasound)	2*	1*
5	Is the safety and efficacy of the SARS-CoV-2 vaccine different in pregnant women compared to non-pregnant women?	4	6
6	What are the effective drugs/procedures to manage women with COVID-19 disease in the 1st, $2^{nd,}$ and 3rd trimester of pregnancy?	5*	5*
7	How can the provision of essential maternal and perinatal health commodities affected by the pandemic be improved?	9	1*
8	In postpartum women with COVID-19, what are the risk and determinants of transmission of SARS-CoV-2 to the newborn by breastfeeding or breastmilk?	6	5*
9	Are pregnant women with complications such as pregnancy-induced hypertension, pre-eclampsia, gestational diabetes, malnutrition, anemia, HIV-1, and malaria at increased risk of SARS-CoV-2 infection or COVID-19 disease compared to women with uncomplicated pregnancies?	7	5*
10	What are the risk and determinants of severe disease or death in pregnant women with SARS-CoV-2 infection or COVID-19 disease (e.g., sociodemographic, immunological/hormonal, microbiota, nutritional)?	8	4

**These questions received the same RPS*

Table 4b. Ranking of the top ten research priority questions on newborn health by income classification of the scorer's country of work

Overall ranking of top ten research questions on newborn health	Ranking by World Bank country income classification	
	Upper and upper middle-income countries	Lower and lower-middle income countries
1 What are the most effective treatment strategies for symptomatic newborns with SARS-CoV-2 infection, especially those with respiratory illness?	4*	1
2 What is the effectiveness of different interventions (e.g., maternal hygiene, facemask use, maternal-newborn separation, etc.) to prevent neonatal acquisition of SARS-CoV-2 in the early postnatal period in the hospital	1*	4

	and at home while breastfeeding, during skin-to-skin contact or kangaroo mother care, especially if the mother has symptomatic COVID-19 disease?		
3	Is SARS-CoV-2 transmissible to healthy infants including newborns of mothers infected with the virus? What are the routes of transmission during in-utero, intrapartum, and postnatal periods (e.g., transplacental, contact with virus present in vaginal or fecal secretions during delivery, breastmilk, etc.)? Does the incidence and severity of infection in the newborns differ by the timing, transmission, or mode of delivery (e.g., cesarean vs. vaginal delivery)?	1*	3*
4	What are the clinical presentations of SARS-CoV-2 infection in newborns? What is the burden of severe COVID-19 disease in newborns?	4*	2
5	What proportion of newborns infected with SARS-CoV-2 presenting for care with hypoxemia receive oxygen therapy, and what is the case fatality rate in this population?	5*	3*
6	What is the most effective way to prevent the transmission of SARS-CoV-2 to preterm newborns receiving kangaroo mother care?	3	5
7	What are the major risk and/or protective factors (e.g., breastfeeding, skin-to-skin care or kangaroo mother care, low birth weight, Bacillus Calmette Guerin or other existing vaccinations, HIV-1 exposure, etc.) for the acquisition of SARS-CoV-2 infection or development of COVID-19 disease or severe disease in newborns of SARS-CoV-2 infected mothers?	5*	6
8	In newborns with severe COVID-19 disease, is the addition of dexamethasone to the standard of care more effective in improving newborn survival compared to the standard of care alone?	9	3
9	Which is the most appropriate diagnostic sample for diagnosis of SARS-CoV-2 infection in newborns (e.g., oral secretion, nasal swab, fecal swab, cord or peripheral blood, etc.?	2	8
10	What is the impact of maternal SARS-CoV-2 infection on newborn health outcomes (e.g., intrauterine growth restriction, prematurity, and birth asphyxia), and how can this be mitigated?	7	7

**These questions received the same RPS*

Table 4c. Ranking of the top ten research priority questions on child and adolescent health by income classification of the scorers' country of work

Overall ranking of top ten research questions on child and adolescent health	Ranking by World Bank country income classification	
	Upper and upper middle-income countries	Lower and lower-middle income countries
1. How can routine vaccination services for children be sustained/improved during the COVID-19 pandemic?	2	1
2. What are the effective and safe strategies for in-class education during the COVID-19 pandemic? What is the attack rate within schools and what interventions (e.g. face masks, physical distancing, hand disinfection/washing, etc.) are effective for prevention of transmission of SARS-CoV-2 in schools, by age/school year and by type of school (e.g. day vs boarding)?	1	5*
3. What are the most effective strategies for communication about the prevention of SARS-CoV-2 infection to adolescents and young adults?	4*	4
4. What are the protective factors, including breastfeeding/feeding practices, maternal vaccination against severe COVID-19 disease in infants, children, and adolescents (0-19 years), and do these differ by age?	7	5*
5. What is the impact of comorbidities (e.g., asthma, diabetes, undernutrition, overweight or obesity, HIV-1 exposure or HIV-1 infection, etc.) in children and adolescents (0-19 years) with SARS-CoV-2 infection on development of disease and on its clinical severity and outcome?	6	7
6. What is the effect of reopening schools on SARS-CoV-2 transmission among different age groups in the general population (based on real-life data)?	4*	5*
7. What is the sensitivity and specificity of SARS-CoV-2 RT-PCR testing in children?	15	3
8. What is the impact of the COVID-19 pandemic on essential child health services (both at a community level and facility level)?	16	2
9. What are the best risk-stratification tools (e.g., Integrated Management of Childhood Illness algorithm, biomarkers, clinical scores, etc.) to triage sick children with suspected SARS-CoV-2 infection or COVID-19 disease, and with sufficiently good performance to predict the severity and outcome?	2	11
10. What is the prevalence of SARS-CoV-2 infection in breastfed vs non-breastfed infants?	12	6

*These questions received the same RPS

Table 4d. Ranking of the top ten research priority questions in cross-cutting/health systems area by the scorers country of work

Overall ranking of top ten research questions in cross-cutting/health systems area	Ranking by World Bank country income classification	
	Upper and upper middle-income countries	Lower and lower-middle income countries
1 What are safe and cost-effective approaches to provide oxygen to pregnant women, newborns, and children who need oxygen in low- and middle-income countries with limited oxygen supply?	7	1
2 What are the barriers to the provision of essential reproductive, maternal, newborn, child, and adolescent health services during the COVID-19 pandemic? What are proven health system approaches/solutions that countries have adapted or implemented to ensure continuity of essential reproductive, maternal, newborn, child, and adolescent health services during the COVID-19 pandemic?	3	2
3 What are the effective health system adaptations to sustain care-seeking and support or resume high-quality essential services for women, newborns, children, and adolescents in COVID-19 and post-COVID-19 contexts?	2	4
4 To what extent are resources (health workers, oxygen support, infection, and prevention control supplies) being diverted from women, newborns, and children or routine services to provide care for COVID-19 disease?	1	8
5 What different models have countries taken to adapt or modify health services for maternal and newborn health during the COVID-19 pandemic and what is the effect of the new adapted or modified or redesigned COVID-19 models of care (e.g. remote antenatal and postnatal services replacing in-person contact) on access, coverage, quality of care and outcomes of mothers and newborns, especially for marginalized populations?	4	6

Table 5. The list of experts who provided research ideas

SN	Name	Organizational affiliation
1	A S M Nawshad Uddin Ahmed	Dhaka Shishu (Children) Hospital, Bangladesh
2	Abigail Kazembe	Kamuzu College of Nursing, Malawi
3	Adegoke Falade	College of Medicine, University of Ibadan and University College Hospital, Ibadan, Nigeria
4	Adnan Bhutta	Pediatric Critical Care Medicine

5	AIDA MUJKIĆ	University of Zagreb, School of Medicine, A.STAMPAR School of Public Health, Croatia
6	Aleksandra WesoÅ‚owska	Laboratory of Human Milk and Lactation Research, Regional Human Milk Bank in Holy Family Hospital, Warsaw Medical University, Poland
7	Alfredo Tagarro	Pediatrics Department. Hospital Universitario Infanta SofÃa. Pediatrics Research Group. Universidad Europea de Madrid. Pediatric Research and Clinical Trials Unit (UPIC). Instituto de InvestigaciÃ³n Sanitaria Hospital 12 de Octubre (IMAS12), Madrid, Spain.
8	Ali A. Abdelmegeid	CEO,Allied Experts for Health Systems, LLC, Egypt
9	Alison Macintyre	WaterAid
10	Aluisio J D Barros	Int Center for Equity in Health, Federal University of Pelots, Brazil
11	Anabelle Bonvecchio	Center for Nutrition and Health Research of the National Institute of Public Health of Mexico
12	Anand Krishnan	AIIMS, New Delhi
13	AndrÃ© Ricardo Araujo da Silva	Professor of Paediatrics, Federal Fluminense University, Brazil
14	Andrew Clarke	Senior Health Advisor; Save the Children UK
15	Angelo Mazza	ASST Papa Giovanni XXIII – Bergamo, Italy
16	Angoulvant	Professor of Pediatric, Paris University
17	Annamarie Saarinen	Newborn Foundation - Research Collaborative; University of Minnesota Pediatric Device Innovation Consortium; Children's Hospital of Fudan University, China
18	Anne Merewood	Boston University; CHEERing
19	Annemieke Brands	WHO Global TB Programme
20	a-philpott@dfid.gov.uk	Senior Health Advisor, Africa Regional, DFID, UK Government
21	Astrid Smeets	Senior Clinical and Medical Affairs Manager at Mother & Child Care - Professional Marketing, Royal Philips
22	Atif Rahman	University of Liverpool, UK; Human Development Research Foundation, Pakistan
23	Audrey Battu	Director at Clinton Health Access Initiative
24	BARRY FINETTE	PROFESSOR OF PEDIATRICS, UNIVERSITY OF VERMONT COLLEGE OF MEDICINE FOUNDER, THINKMD PBC
25	Braishna Jafar	Society for the Protection of the Rights of Children Pakistan
26	Brooke Rogers	King's College London
27	Camille Raynes-Greenow	The University of Sydney
28	Carina King	Karolinska Institutet
29	Carsten Krager	University of Witten-Herdecke
30	Casie Tesfai	International Rescue Committee
31	Catherine Herba	UniversitÃ© du QuÃ©bec Ã MontrÃ©al and Centre de Recherche du CHU Sainte Justine

32	Catherine Pitt	London School of Hygiene & Tropical Medicine
33	Catherine S Birken	Professor of Paediatrics, University of Toronto, Senior Scientist, SickKids Research Institute
34	Cesar Victora	Federal University of Pelotas
35	Charles Shey Wiysonge	South African Medical Research Council
36	Charlotte E Warren	Population Council
37	Pooja Sripad	Population Council
38	Cindy McWhorter	UNICEF
39	Concha Bonet De Luna	SERMAS, EMT SPAIN (AECID)
40	Daniel Martinez Garcia	Paediatric Advisor, Médecins Sans Frontières
41	Daniel Raiten	Program Director-Nutrition, NICHD/NIH
42	Dr Azizi	work in RMNCAH Directorate as Advisor
43	AYEDE Adejumoke Idowu	Department of Paediatrics, College of Medicine University of Ibadan & Consultant Paediatrician University College Hospital Ibadan.
44	Ajay Kumar Khera	Country Representative<br>India Country Office
45	Lilian Kiapi	International Rescue Committee
46	Mariyam Jenyfa	Health Protection Agency, Ministry of Health
47	Marzia Lazzerini	WHO Collaborating Centre for Maternal and Child Health , Institute for Maternal and Child Health IRCCS Burlo Garofolo
48	Nagiba A. Abdulghani AlShawafi	self-employed
49	Naila Ghazi	MoPH
50	Rabeya Khatoon	Ex-WHO, now Self-employed
51	Reeta Raisily	Indian Council of Medical Research, Ansari Nagar, New Delhi
52	Tara Mangal	Imperial College London
53	Asma Badar	Save the Children
54	Daniel Murokora	Ministry of Health
55	Dhana Raj Aryal	Ishan Children and Maternity Hospital, kathmandu
56	Doug McMillan	Professor Emeritus University of Calgary and Professor Emeritus Dalhousie University, Halifax, Canada
57	Fahad A Al Aql	Saudi MOH
58	Homaira Abawi	N/A
59	Oya Zeren Afsar	Health Specialist, HPV Coordinator, Health Section, UNICEF HQ New York
60	Praveen Kumar	Division of Neonatology, Department of Pediatrics, Post Graduate Institute of Medical Education and Research, Chandigarh, India
61	Vishwajeet Kumar	Community Empowerment Lab
62	Massouma Neghat	RMNCAH Directorate, Ministry of Public Health (MoPH), Afghanistan

63	Ebunoluwa Adejuyigbe	Obafemi Awolowo University, Ile-Ife, Nigeria
64	Ecaterina Stasii,	Professor, State Medical and Pharmaceutical University after Nicolae Testemitanu, Department of Paediatrics
65	Elizabeth McClure	Research Triangle Institute
66	Els LIM Duval	Clinical Head PICU Antwerp University Hospital Antwerp
67	Emma Zaja	Department for International Development, UK
68	Eric A.F. Simoes	University of Colorado school of medicine; Samshoma Medical Research Inc.
69	Eric D McCollum	Johns Hopkins School of Medicine
70	Erica Corbett	Consultant in MNCH research; affiliated with Dalhousie University, Canada
71	Fabian Esamai	Moi University
72	Fatma AL Hinai	Ministry of Health
73	George Patton	University of Melbourne, Murdoch Children's Research Institute, Australia
75	Greta Mino	Hospital Alcivar /CEIIP
76	Gunta Lazdane	Riga Stradins University
77	Halvor Sommerfelt	CISMAC (www.cismac.org), University of Bergen
78	Hamish Graham	Centre for International Child Health, MCRI, University of Melbourne
79	Hande Harmanci	WHO Representative, Azerbaijan
80	Hani W Fawzi	FIGO Director of projects
81	Harriet Birungi	Population Council
82	Harshpal Singh Sachdev	Senior Consultant Pediatrics and Clinical Epidemiology, Sitaram Bhartia Institute of Science and Research, New Delhi
83	He-Feng Huang	Shanghai Jiao Tong University
84	Henia Dakkak	United Nations Population Fund
85	Hirondina Cucubica	UNICEF
86	Israel Amirav	Tel aviv Medical Center Tel Aviv Israel
87	Jamela Al-Raiby	WHO
88	Jeannette Wadula	Clinical Microbiologist, Infection Prevention Practitioner. National Health Laboratory Services, Dept of Clinical Microbiology & Infectious Diseases, University of Witwatersrand
89	Joan Lombardi.	Director Early Opportunities and Senior Scholar, Center for Child and Human Development, Georgetown University
90	Joao Paulo Souza	University of SÃ£o Paulo
91	Jocelyn Chan	Murdoch Children's Research Institute
92	Jose Carlos Martines	CISMAC, University of Bergen, Norway
93	Joseph de Graft-Johnson	Save the Children
94	Juan Angel Rivera Dommarco	National Institute of Public Health of Mexico

95	Juan M. Lozano	Professor, Department of Translational Medicine, College of Medicine, Florida International University, Miami, Florida, USA
96	Judd Walson,	Vice Chair and Professor, Department of Global Health, University of Washington
97	Justien Dol	Dalhousie University
98	Kanar Wael Qadi (Ms.)	Health and Nutrition Specialist<br>UNICEF State of Palestine | 13 Faydi El-Alami Street, P.O.Box: 25141, East Jerusalem
99	Karel allegaert	Academic researcher, KU Leuven Belgium and Erasmus MC, Rotterdam, the Netherlands
100	Kate Gilroy	John Snow, Inc; MOMENTUM Knowledge Accelerator
101	Kathleen M. Rasmussen	Cornell University
102	Keiko Osaki	Japan International Cooperation Agency
103	Kevin Baker	Malaria Consortium
104	Kirsty Le Doare	MRC/UVRI @LSHTM UGanda
105	Kristina Keitel	Swiss Tropical and Public Health Institute; University Children's Hospital Bern, Switzerland
106	L Booij	CHU Sainte Justine
107	Laura A Burnham	Center for Health, Equity, Education, & Research (CHEER) at Boston Medical Center
108	Lay-Myint Yoshida	Department of Pediatric Infectious Diseases, Institute of Tropical Medicine, Nagasaki University
109	Lee Pyne-Mercier	Bill & Melinda Gates Foundation
110	Lindsay Keir	Wellcome Trust and University of Bristol
111	Lindsay Stark	Associate Professor<br>Washington University in St. Louis
112	Lisa Noguchi,	Jhpiego and Johns Hopkins University
113	Louise Tina Day	London School of Hygiene & Tropical Medicine
114	Lynne Jones	Honorary consultant child and Adolescent psychiatrist, South London and Maudsley NHS foundation trust
115	Lynne M. Mofenson	Senior HIV Technical Advisor, Elizabeth Glaser Pediatric AIDS Foundation
116	Maher Aboumayaleh	Ù • Aga Khan Health Services / Syria
117	Mamdouh Wahba	Egyptian Family Health Society, Egyptian Society for Adolescent Medicine, Arab Coalition for Adolescent Health & Med.
118	Maria Carmen COllado	CSIC, spain
119	Marie McGrath	Technical Director, Emergency Nutrition Network
120	MARION KOSO-THOMAS	National Institute of Health
121	Maristela Monteiro	Pan American Health Organization
122	Mark Ansermino	The University of British Columbia
123	Mark van Ommeren	Unit Head, mental health

124	Marta Nunes	DST/NRF:Vaccine Preventable Diseases<br>Respiratory and Meningeal Pathogens Research Unit<br>CH Baragwanath Hospital<br>New Nurses Home, West Wing, 1st Floor
125	Marina Melkumova	Arabkir Medical Centre-Institute of Child and Adolescent Health
126	Mary Kinney	School of Public Health, University of the Western Cape
127	Mary Penny	Instituto de Investigacion Nutricional, Lima, Peru
128	Maxine Lashley	Barbados Community College
129	Meena Gandhi	Department for International Development
130	Meinzer Ulrich	Department of General Pediatrics, Pediatric Internal Medicine, Rheumatology and Infectious Diseases, National Referee Centre for Rare Pediatric Inflammatory Rheumatisms and Systemic Auto-immune diseases RAISE, Robert Debré University Hospital, Assistance Publique-Hôpitaux de Paris, Paris, France
131	Melania Maria Ramos de Amorim	Instituto de Medicina Integral Prof. Fernando Figueira (IMIP) and Instituto Paraibano de Pesquisa Professor Joaquim Amorim Neto (IPESQ)
132	Melissa Bartick	Mt Auburn Hospital, Harvard Medical School
133	Michael Head	University of Southampton, UK
134	Michel PacquÃ©	JSI
135	Mike English	KEMRI-Wellcome Trust, Kenya
136	Moazzam Ali	WHO
137	Mohammad Salim Bahadury	RMNCAH Directorate, Ministry of Public Health (MoPH), Afghanistan
138	Montasser Kamal	Program Leader, Maternal and Child Health, International Development Research Centre
139	Mr. Khalil Ahmed	Manager Research & Communication, Society for the Protection of the Rights of the Child (SPARC)
140	Munir	Track20 officer
141	n.french@liverpool.ac.uk	University of Liverpool/Malawi Liverpool Wellcome Trust
142	Nabila SEDDIKI	Paris-Est Créteil University and INSERM
143	Nada Ahmed	Public Health Consultant
144	Nafissa Mohamed Rashid Bique Osman	Consultant Obs/Gyn Hospital Central de Maputo and Professor at Universidade Eduardo Mondlane
145	Nana A. Y. Twum-Danso, MD, MPH	The Rockefeller Foundation
146	Natalia Largaespada Beer	Ministry of Health, Belize
147	Naziha Bahadur Ahmadi	Government /Ministry of Public Health of Afghanistan
148	Nieneke van den Akker	Cordaid
149	Ola Didrik Saugstad	Department of Pediatric Research, University of Oslo, Norway
150	Omer ERDEVE	Ankara University School of Medicine, Department of Pediatrics, Division of Neonatology, Ankara, Turkey
151	Oya Zeren Afsar	Health Specialist, Health Section, UNICEF NYHQ

152	Pablo Rojo	Hospital 12 de Octubre
153	Pascale Ondoa	African Society for Laboratory Medicine
154	Patricia Jodrey	USAID Washington
155	Paul Bolton	Johns Hopkins Bloomberg School of Public Health
156	peter Azzopardi	burnet institute
157	Philippe Van de Perre	University of Montpellier
158	phuongty832000@gmail.com	Da Nang hospital for Women and Children, Paediatrician
159	Portia Mutevedzi	Vaccines and Infectious Diseases Analytics Research Unit (MRC- VIDA)
160	Debby Bogaert	Professor,University of Edinburgh
162	Shrijana Shrestha	Professor,Patan Academy of Health Sciences, Nepal
163	Soofia Khatoon	Professor,Academic Director and Head of Paediatrics, Center for Woman and Child Health, Dhaka, Bangladesh
164	Tippawan Liabsuetrakul	Professor,Faculty of Medicine, Prince of Songkla University, Hat Yai, Songkhla, Thailand
165	Judith Rankin	Professor, Population Health Sciences Institute, Newcastle University
166	PKirsty Donald	Professor,University of cape Town
167	Olena Starets	Professor,National Medical University, Odessa, Ukraine
168	Prudence Hamade	p.hamade@malariaconsortium.org
169	Qalab abbas	Aga Khan University
170	Quique Bassat	ISGlobal, Barcelona, Spain
171	Rashed Shah	Save the Children US
172	Rebecca Grais	Epicentre
173	Rebecca Richards-Kortum	Rice University
174	Rizwan Ullah	MoPH Afghanistan employee
175	robert black	Johns Hopkins University
176	Robert Pattinson	SAMRC/UP Maternal and Infant Health Care Strategies unit
178	Ruba Samain	Employed at the Queen Rania Foundation for Education and Development
179	Rusidah Selamat	Nutrition Division, Ministry of Health Malaysia, Putrajaya
180	Ruth Davidge	neonatal Nurses association of Southern Africa
181	Sabrina Kitaka	Makerere University College of Health Sciences
182	Sarah Williams	Save the Children, UK
183	Senait Kebede	Adjunct Associate Professor , Global Health, Emory University , Leadr Instruct AMR , Africa CDC/Emory
184	Shabir Madhi	DST/NRF:Vaccine Preventable Diseases<br>Respiratory and Meningeal Pathogens Research Unit<br>CH Baragwanath Hospital<br>New Nurses Home, West Wing, 1st Floor

185	Sharon Goldfeld	Murdoch Children's Research Institute/Centre for Community Child Health
186	Shelley McGuire	University of Idaho (USA)
187	Shukrullah Shakir	MoPH/RMNCAH
188	Silvia Minozzi	Department of Epidemiology, Lazio Regional Health Service , Rome, Italy
189	Soo Downe	Professor of Midwifery, THRIVE Centre, University of Central Lancashire, UK
190	Stephanie Jones	DST/NRF:Vaccine Preventable Diseases, Respiratory and Meningeal Pathogens Research Unit, CH Baragwanath Hospital
191	Suellen Miller	University of California, San Francisco
192	SYLLA Mariam	CHU Gabriel Toure,
193	Sam Schoenmakers	Subdivision of Obstetrical and fetal medicine, Department of Obestetrics and Gynaecology, Erasmus MC University Medical Center, Rotterdam, The Netherlands
194	Sylvia Ley	Tulane University School of Public Health and Tropical Medicine
195	Takashi Suzuki	Obstetrics/Gynecologist, Seirei Hamamatsu General Hospital
196	Theresa Betancourt	Boston college
197	Tim Colbourn	UCL Institute for Global Health
198	Ting Shi	University of Edinburgh
199	Tisungane Mvalo	University of North Carolina Project Malawi (UNCPM)
200	Tobias Alfven	Karolinska Institutet
201	Tor Strand	University of Bergen
202	ValÃ©riane Leroy	Inserm, Reacting, ANRS, France
203	Wammanda Daniel Robinson	Ahmadu Bello University/Ahmadu Bello University Teaching Hospital, Zaria
204	William Checkley	Johns Hopkins University
205	William Cherniak	Bridge to Health Medical and Dental, Rocket Doctor and the University of Toronto
206	Winifride Mwebesa	Save the Children
207	Yan-Ting Wu	Shanghai Jiao Tong University
208	Yap-Seng Chong	Yong Loo Lin School of Medicine, National University of Singapore
209	Yeva Movsesyan	Arabkir MC-Institute of Child and Adolescent Health
210	Yogesh Kumar Shashikanth	Associate Professor, Department of Community Medicine, Project Coordinator, JNMC Women's and Children's Health Research Unit (WHO Collaborating Centre for Maternal and Perinatal Health), J.N.Medical College, KAHER, Belagavi, Karnataka, India
